# Thailand's national strategic plan on antimicrobial resistance: progress and challenges

**DOI:** 10.2471/BLT.20.280644

**Published:** 2021-07-01

**Authors:** Nithima Sumpradit, Suriya Wongkongkathep, Kumthorn Malathum, Noppavan Janejai, Wantana Paveenkittiporn, Thitipong Yingyong, Teerasak Chuxnum, Amornrat Vijitleela, Phairam Boonyarit, Chutima Akaleephan, Weerawat Manosuthi, Varaporn Thienthong, Julaporn Srinha, Supaporn Wongsrichai, Thitiporn Laoprasert, Pornpimon Athipunyakom, Nathaya Kriengchaiyaprug, Kingdao Intarukdach, Sukanya Numsawad, Nuntiya Somjetanakul, Sirima Punnin, Niyada Kiatying-Angsulee

**Affiliations:** a Food and Drug Administration, Ministry of Public Health, 88/24 Tiwanon Road, Mueng District, Nonthaburi, Thailand 11000.; b Office of Permanent Secretary, Ministry of Public Health, Nonthaburi, Thailand.; c Faculty of Medicine, Ramathibodi Hospital, Mahidol University, Bangkok, Thailand.; d Department of Medical Sciences, Ministry of Public Health, Nonthaburi, Thailand.; e Department of Disease Control, Ministry of Public Health, Nonthaburi, Thailand.; f Department of Medical Services, Ministry of Public Health, Nonthaburi, Thailand.; g Department of Livestock Development, Ministry of Agriculture and Cooperatives, Bangkok, Thailand.; h Department of Fisheries, Ministry of Agriculture and Cooperatives, Bangkok, Thailand.; i Department of Agriculture, Ministry of Agriculture and Cooperatives, Bangkok, Thailand.; j Department of Health Service Support, Ministry of Public Health, Nonthaburi, Thailand.; k Department of Pollution Control, Ministry of Natural Resources and Environment, Bangkok, Thailand.; l Drug System Monitoring and Development Centre, Chulalongkorn University, Bangkok, Thailand.

## Abstract

Antimicrobial resistance is a serious threat that affects all countries. The Global Action Plan on antimicrobial resistance and the United Nations Political Declaration on antimicrobial resistance set standards for countries to resolve antimicrobial resistance challenges under the One Health approach. We assess progress and challenges in implementing Thailand’s national strategic plan on antimicrobial resistance 2017–2022, discuss interim outcomes and share lessons learnt. Major progress includes: establishing a national governance mechanism that leads high-impact policy on antimicrobial resistance and consolidates actions and multisectoral collaboration; creating a monitoring system and platform to track implementation of the strategic plan; and converting strategies of the strategic plan into actions such as controlling the distribution and use of antimicrobials in humans and animals. Interim results indicate that antimicrobial consumption in animals has nearly halved (exceeding the national goal of a 30% reduction) whereas other goals have not yet reached their targets. We have learnt that elevating antimicrobial resistance to high-level visibility and establishing a national governance mechanism is an important first step, and a monitoring and evaluation system should be developed in parallel with implementation. Securing funds is crucial. Policy coherence is needed to avoid duplication of actions. Highly ambitious goals, although yet to be achieved, can advance actions beyond expectations. Political commitment and collaboration across different sectors will continue to play important roles but might not be sustained without a well-designed governance structure to support long-term actions to address antimicrobial resistance.

## Résumé

La résistance aux antimicrobiens fait peser une sérieuse menace sur la planète tout entière. Le Plan d'action mondial pour combattre la résistance aux antimicrobiens ainsi que la Déclaration politique des Nations Unies sur la résistance aux agents antimicrobiens ont défini des normes pour les pays, afin qu'ils puissent faire face aux enjeux liés à la résistance aux antimicrobiens selon l'approche «One Health». Nous avons évalué les progrès et défis de la mise en œuvre du plan stratégique national de la Thaïlande en la matière pour 2017–2022, mais aussi discuté des résultats provisoires et partagé les enseignements tirés. Parmi les principaux progrès accomplis figurent l'établissement d'un mécanisme de gouvernance national pour mener une politique à impact élevé sur la résistance aux antimicrobiens, renforcer les actions et favoriser la collaboration intersectorielle; la création d'un système de surveillance et d'une plateforme pour suivre la mise en œuvre du plan stratégique; et enfin, la conversion des stratégies du plan en actions telles que le contrôle de la distribution et de l'usage des antimicrobiens chez les humains et les animaux. Les résultats provisoires indiquent que la consommation d'antimicrobiens chez les animaux a diminué de moitié (ce qui est supérieur à l'objectif national d'une réduction de 30%), tandis que les autres objectifs n'ont pas encore été atteints. Nous avons constaté qu'accroître la visibilité de la résistance aux antimicrobiens et instaurer un mécanisme de gouvernance national constituaient des étapes cruciales, et qu'un système de surveillance et d'évaluation devait être développé parallèlement à la mise en œuvre. L'obtention de financements est elle aussi essentielle. Une politique cohérente est nécessaire pour éviter de multiplier les actions similaires. Fixer des objectifs très ambitieux, même s'ils ne sont pas encore atteints, permet en outre de faire progresser les actions au-delà des attentes. Enfin, l'engagement politique et la collaboration entre différents secteurs continueront à jouer un rôle prépondérant, mais ne pourront peut-être pas se poursuivre sans une structure de gouvernance bien conçue, capable de soutenir des actions à long terme visant à remédier à la résistance aux antimicrobiens.

## Resumen

La resistencia a los antimicrobianos es una grave amenaza que afecta a todos los países. El Plan de Acción Mundial sobre la resistencia a los antimicrobianos y la Declaración Política de las Naciones Unidas sobre la resistencia a los antimicrobianos establecen normas para que los países resuelvan los problemas de resistencia a los antimicrobianos en el marco del enfoque «Una única salud». Evaluamos los avances y los desafíos en la aplicación del plan estratégico nacional de Tailandia sobre la resistencia a los antimicrobianos 2017-2022, analizamos los resultados provisionales y compartimos las lecciones aprendidas. Entre los principales avances se encuentran: el establecimiento de un mecanismo de gobernanza nacional que lidera la política de alto impacto sobre la resistencia a los antimicrobianos y consolida las acciones y la colaboración multisectorial; la creación de un sistema de seguimiento y una plataforma para seguir la aplicación del plan estratégico; y la conversión de las estrategias del plan estratégico en acciones como el control de la distribución y el uso de antimicrobianos en humanos y animales. Los resultados provisionales indican que el consumo de antimicrobianos en animales se ha reducido casi a la mitad (superando el objetivo nacional de una reducción del 30 %), mientras que otros objetivos aún no han alcanzado sus metas. Hemos aprendido que elevar la resistencia a los antimicrobianos a una visibilidad de alto nivel y establecer un mecanismo de gobernanza nacional es un primer paso importante, y que debe desarrollarse un sistema de seguimiento y evaluación en paralelo a la implementación. Asegurar los fondos es crucial. La coherencia política es necesaria para evitar la duplicación de acciones. Unos objetivos muy ambiciosos, aunque todavía no se hayan alcanzado, pueden hacer avanzar las acciones más allá de las expectativas. El compromiso político y la colaboración entre los distintos sectores seguirán desempeñando un papel importante, pero podrían no mantenerse sin una estructura de gobernanza bien diseñada que apoye las acciones a largo plazo para hacer frente a la resistencia a los antimicrobianos.

## ملخص

تعتبر مقاومة مضادات الميكروبات تهديدًا خطيرًا يؤثر على كل البلدان. وضعت كل من خطة العمل العالمية بشأن مقاومة مضادات الميكروبات، والإعلان السياسي للأمم المتحدة بشأن مقاومة مضادات الميكروبات، معايير للبلدان لحل تحديات مقاومة مضادات الميكروبات في إطار نهج One Health (وان هيلث). نحن نقوم بتقييم التقدم والتحديات في تنفيذ الخطة الاستراتيجية الوطنية لتايلند بشأن مقاومة مضادات الميكروبات خلال الفترة من 2017 إلى 2022، ومناقشة النتائج المؤقتة وتبادل الدروس المستفادة. يشمل التقدم الرئيسي ما يلي: إنشاء آلية حوكمة وطنية تقود سياسة عالية التأثير بشأن مقاومة مضادات الميكروبات، ودعم الإجراءات والتعاون متعدد القطاعات؛ وإنشاء نظام ومنصة مراقبة لتتبع تنفيذ الخطة الاستراتيجية؛ وتحويل استراتيجيات الخطة الاستراتيجية إلى إجراءات مثل التحكم في توزيع واستخدام مضادات الميكروبات في البشر والحيوانات. تشير النتائج المؤقتة إلى أن استهلاك مضادات الميكروبات في الحيوانات قد انخفض إلى النصف تقريبًا (متجاوزًا الهدف الوطني البالغ 30%) في حين أن الأهداف الأخرى لم تحقق غاياتها بعد. لقد تعلمنا أن رفع مقاومة مضادات الميكروبات إلى الرؤية عالية المستوى، وإنشاء آلية حوكمة وطنية، هي خطوة أولى مهمة، كما يجب تطوير نظام للرصد والتقييم بالتوازي مع التنفيذ. كذلك، فإن تأمين الأموال أمر بالغ الأهمية. هناك حاجة لتماسك السياسات لتجنب ازدواجية الإجراءات. ويمكن للأهداف الطموحة للغاية، على الرغم من أنها لم تتحقق بعد، أن ترتقي بالإجراءات لما يتجاوز التوقعات. سوف يستمر الالتزام السياسي والتعاون عبر مختلف القطاعات، في لعب أدوار مهمة، ولكنهما قد لا يدومان دون هيكل حوكمة جيد التصميم لدعم الإجراءات طويلة الأجل لمعالجة مقاومة مضادات الميكروبات.

## 摘要

抗生素耐药性是一个严重影响各个国家的威胁因素。《抗生素耐药性问题全球行动计划》和《联合国抗生素耐药性政治宣言》为各国在“健康全覆盖”方针指导下解决抗生素耐药性挑战确立了标准。我们评估泰国自 2017 年至 2022 年在抗生素耐药性国家战略计划实施方面所取得的进展和面临的挑战，同时还讨论所取得的阶段性成果并分享经验教训。主要进展包括：建立国家治理机制，引导制定在抗生素耐药性问题方面具有重大影响力的政策，同时开展多部门协作的联合行动；建立监控系统和平台以追踪战略计划的实施情况；以及将战略计划落实到实际行动，例如管控抗生素在人类和动物群体中的分配和使用。所取得的阶段性成果表明，动物群体中的抗生素用药量几乎减半（超出国家目标的 30%），不过其它目标尚未达到所设定的目标值。我们总结发现，提高抗生素耐药性的透明度和建立国家治理机制是重要的第一步，而且应该在实施计划的同时建立监测和评估体系。有保障的资金也是至关重要的因素。需要保持政策协调一致，以避免重复的行动。所设定的宏伟目标虽然尚未实现，但可以激发超出预期的行动力。不同部门之间坚定的政治承诺和协作将继续发挥重要的作用，但是如果没有合理而有效的治理架构来为应对抗生素耐药性问题的长期行动提供支持，那么这些承诺和协作可能难以为继。

## Резюме

Устойчивость к антимикробным препаратам является серьезной угрозой, которая затрагивает все страны. Глобальный план действий по борьбе с устойчивостью к антимикробным препаратам и Политическая декларация Организации Объединенных Наций по борьбе с устойчивостью к антимикробным препаратам устанавливают стандарты для стран в части решения проблем устойчивости к антимикробным препаратам в рамках подхода «Единое здоровье». Авторы оценивают прогресс и проблемы в реализации национального стратегического плана Таиланда по борьбе с устойчивостью к антимикробным препаратам на 2017–2022 годы, обсуждают промежуточные результаты и делятся полученными уроками. К значительному прогрессу относятся: создание национального механизма управления, который руководит высокоэффективной политикой в отношении устойчивости к антимикробным препаратам и объединяет действия и многосекторальное сотрудничество, создание системы мониторинга и платформы для отслеживания выполнения стратегического плана и преобразование запланированных стратегий в действия, такие как контроль распространения и использования антимикробных препаратов среди людей и животных. Промежуточные результаты показывают, что потребление антимикробных препаратов животными сократилось почти вдвое (превысив национальную цель в 30%), в то время как другие цели еще не достигли нужных показателей. Мы поняли, что привлечение как можно более широкого внимания к проблеме устойчивости к антимикробным препаратам и создание национального механизма управления являются важным первым шагом, а система мониторинга и оценки должна разрабатываться параллельно с внедрением. Обеспечение финансирования имеет решающее значение. Необходимо проводить последовательную политику во избежание дублирования действий. Весьма амбициозные цели, хотя они еще не достигнуты, могут способствовать тому, что исполнители будут действовать, превосходя предъявляемые ожидания. Политическая воля и сотрудничество между различными секторами продолжат играть важную роль, но вряд ли смогут сохранить свое действие без хорошо продуманной структуры управления, поддерживающей долгосрочные действия по борьбе с устойчивостью к антимикробным препаратам.

## Introduction

Antimicrobial resistance is a prominent global threat that jeopardizes the health of humans and animals, and the economy of countries. Antimicrobial resistance also threatens global health security and hampers the achievement of the sustainable development goals (SDGs).^1^^,^^2^ In 2019, the World Health Organization (WHO) listed antimicrobial resistance in its top-10 global public health threats facing humanity^3^ and in 2020 antimicrobial resistance became a new indicator for the SDGs.^4^ Addressing antimicrobial resistance requires consolidated multisectoral actions under the One Health approach – a multisectoral and multidisciplinary collaboration that connects human, animal and environmental health sectors to improve the health of all.^5^ The 2015 Global Action Plan on antimicrobial resistance^6^ and the 2016 United Nations Political Declaration on antimicrobial resistance^7^ serve as standards for countries, development partners and relevant stakeholders to develop their action plans and align actions in tackling antimicrobial resistance.

Antimicrobial resistance affects every country,^8^ including upper-middle-income countries such as Thailand. Thus, in 2016, the government of Thailand endorsed the national strategic plan on antimicrobial resistance 2017–2021, which was recently extended to 2022. The plan takes the Global Action Plan on antimicrobial resistance into account and aligns political declarations with the national context. The plan included a set of ambitious goals together with six strategies for achieving them: (i) antimicrobial resistance surveillance; (ii) regulation of antimicrobial distribution; (iii) antimicrobial resistance containment in humans; (iv) antimicrobial resistance containment in agriculture and animals; (v) public awareness-raising; and (vi) governance mechanisms.^9^

At the outset, the government of Thailand established two key strategic foundations for implementing the plan (Box 1). First, a national governance mechanism was created as a political platform to strengthen multisectoral collaboration under the One Health approach. This mechanism aimed to bring technical issues on antimicrobial resistance into the political arena by establishing a national policy committee on antimicrobial resistance. The committee serves to take forward high-impact policy initiatives on antimicrobial resistance in a coordinated manner among the responsible agencies.

Box 1 **Two strategic foundations for implementing Thailand’s national strategic plan on antimicrobial resistance 2017–2022**

**Establishment of a national governance mechanism**

Thailand’s national policy committee on antimicrobial resistance (chaired by the deputy prime minister) is a high-level, multistakeholder steering panel from government agencies, professional organizations, civil society organizations, private sector and academia aiming to steer and lead high-impact policy on antimicrobial resistance. Five subcommittees under the National Policy Committee on antimicrobial resistance were appointed to supervise and guide the implementation of individual strategies. A strategic coordinating group consisting of secretary teams, experts and relevant partners provides a horizontal mechanism for coordination across the plan.

**Development of a scientific platform to generate evidence to guide policy**

Antimicrobial resistance is one of six priority programmes in the World Health Organization (WHO) country strategy for Thailand, 2017–2021. The programme pools funds provided by the public health ministry, the Thai Health Promotion Foundation, the National Health Security Office, the Health Systems Research Institute and WHO Thailand.^10^ The programme serves as the scientific arm of the National Policy Committee on antimicrobial resistance and is overseen by Thailand’s Food and Drug Administration together with the Health Intervention and Technology Assessment Program Foundation mutually. Each year, the programme provides grants for several projects linked to Thailand’s national strategic plan on antimicrobial resistance. Key research partners include, but are not limited to, the International Health Policy Program, agencies from the public health ministry, the agriculture and cooperatives ministry and academia. Essential evidence generated through the programme includes baseline data of the national goals;^11^^,^^12^ the midterm review of implementation of Thailand’s national strategic plan on antimicrobial resistance;^13^^,^^14^ and a study on mapping and prioritizing antimicrobial resistance research.^15^ Monitoring and evaluation systems include Thailand’s system for surveillance of antimicrobial consumption to monitor consumption and distribution of antimicrobials for human and animal use.^11^

Second, a scientific platform was developed to generate evidence to guide policy development, implementation and monitoring. Previously, only fragmented data on antimicrobial resistance and antimicrobial use were available for the human, livestock and food sectors, with limited or no data on the aquaculture, plant, companion animal and environment sectors.^13^ During 2017–2021, antimicrobial resistance was listed as a priority area in the WHO country cooperation strategy for Thailand. This cooperation strategy utilizes a pooled funding mechanism and leverages the social and intellectual capital of country cooperation strategy partners in developing the required monitoring and evaluation systems and generating the evidence needed to support the plan.^10^^,^^16^

We outline here the progress and challenges of implementing Thailand’s national strategic plan on antimicrobial resistance from 2017 until the middle of 2021. We also discuss interim outcomes and share lessons learnt. 

## Progress and challenges

### Surveillance system

**Fig. 1:**
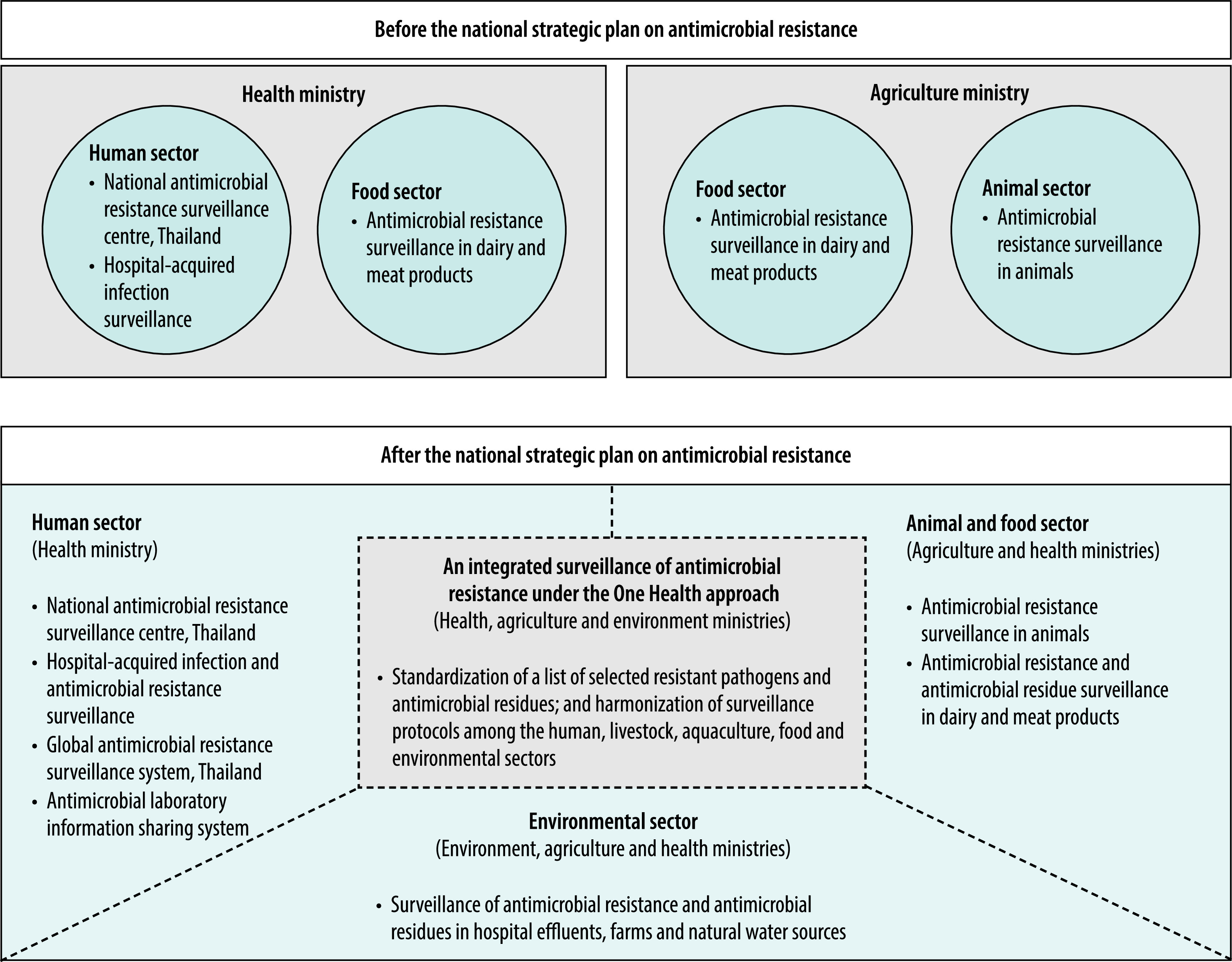
Changes to the antimicrobial resistance surveillance under the One Health approach in Thailand

Note: Before the national strategic plan on antimicrobial resistance, antimicrobial resistance surveillance covered only humans, animals and food, not the environmental sector. It is operated by the health ministry and the agriculture ministry without a coordinating mechanism. After the plan implementation, the health, agriculture and environment ministries successfully established an integrated surveillance network of antimicrobial resistance under the One Health approach. This network not only strengthens the laboratory capacity such as standardizing laboratory protocols, it allows cross-learning and enhances collaboration across different sectors working on antimicrobial resistance under the One Health approach.

Strategy 1 of the national strategic plan was to establish an antimicrobial resistance surveillance system under the One Health approach (Box 2). Thailand has been able to strengthen its existing antimicrobial resistance surveillance systems (such as in the human, livestock and food sectors) and has developed new surveillance systems, for example, in aquaculture and the environment. A key achievement is the establishment of a national integrated surveillance of antimicrobial resistance network led by the health, agriculture and environment ministries. Our team developed new standard protocols on antimicrobial resistance surveillance to monitor the prevalence and relationship of targeted bacteria across the human, livestock, aquaculture, food and environmental sectors (Fig. 1). Staff from different ministries have provided technical and facility support to overcome the limitations of the microbiology laboratories in the network.

Box 2 **Antimicrobial resistance surveillance system: Strategy 1 of Thailand’s national strategic plan on antimicrobial resistance 2017–2022**

**Strategy**

Antimicrobial resistance surveillance system using a One Health approach.

**Achievements**

- Establishing a national integrated surveillance network of antimicrobial resistance under the One Health approach. The process was led by the public health ministry, agriculture and cooperatives ministry and natural resources and environment ministry. The strategy aimed to standardize and harmonize antimicrobial resistance surveillance protocols across the human, animal, food and environmental sectors. The first report on integrated surveillance of antimicrobial resistance under the One Health approach was planned for 2022.
- Strengthening the capacity and network of microbiology laboratories in the human, animal (livestock and aquaculture), food and environmental sectors.
- Implementing antimicrobial resistance surveillance of extended spectrum β-lactamase *Escherichia coli* under WHO’s Tricycle project. This project encourages countries to build national integrated, multisectoral surveillance systems for antimicrobial resistance using extended β-lactamase producing *E. coli* as a simple indicator in the human, food (animal) and environmental sectors.
- Implementing the WHO global antimicrobial resistance surveillance system in 10 hospitals as sentinel sites representing each health region.
- Integrating antimicrobial resistance into surveillance of health-care-associated infections in hospitals.
- Generating a national epidemiological surveillance and response system of antimicrobial resistance, including antimicrobial resistance case reports for five important emerging antimicrobial-resistant pathogens.
- Creating an antimicrobial resistance laboratory-based surveillance system as a prototype of a single-portal hospital database on antimicrobial resistance.
- Improving the capacity for microbiology laboratory surveillance.
- Strengthening epidemiological capacity on surveillance and response to important emerging antimicrobial-resistant pathogens by enhancing the skills of infection control nurses, physicians, pharmacists, laboratory technicians and public health officers.
- Advancing Thailand’s national antimicrobial resistance surveillance centre system. The aim was to illustrate the national and local patterns and trends of antimicrobial resistance antibiograms across 12 health regions and among hospitals and to allow for local comparisons.
- Establishing a national surveillance programme for antimicrobial resistance in food-producing animals (such as broiler chickens and pigs). Developing a framework for antimicrobial resistance surveillance.

**Strengths**

- Existence of a national antimicrobial resistance surveillance centre and a national surveillance programme for antimicrobial resistance in food-producing animals. This structure serves to engage relevant microbiology laboratories to further advance antimicrobial resistance surveillance under the One Health approach.
- Strong technical leadership in laboratory surveillance of antimicrobial resistance.

**Challenges**

- Fragmentation of databases within and across sectors.
- Surveillance data on antimicrobial resistance and antimicrobial residues in the non-human sector can sometimes be sensitive as they relate to the economy. There is therefore a need to create mutual understanding across multiple sectors with different interests to facilitate data-sharing and creating an integrated surveillance of antimicrobial resistance under the One Health approach.

**Ways forward**

- Incorporating surveillance of antimicrobial residues and antimicrobial consumption as an integral part of the national integrated system of antimicrobial resistance surveillance under the One Health approach.
- Developing a single portal for surveillance of antimicrobial resistance-associated morbidity in hospitals.

WHO: World Health Organization.

In the human sector, antimicrobial resistance surveillance systems are complex because each existing system has its own organizational mandate. Developing a new system to capture antimicrobial resistance morbidity could have generated additional workload and created another separate system. Instead, we redesigned the data flow of these existing surveillance systems to form a data pool for analysis (Fig. 1).

We learnt that modifying antimicrobial resistance surveillance systems and synchronizing the data is difficult when these systems are established independently by each agency (such as in hospitals). However, data fragmentation can be prevented or mitigated in surveillance systems that are newly developed or have not yet been fully established (such as those in non-human sectors). 

### Regulation of distribution

Strategy 2 of the national strategic plan was the regulation of antimicrobial distribution (Box 3). Government legislation in Thailand allows antibiotics to be dispensed by a licensed pharmacist without a prescription.^18^ Unfortunately, this arrangement cannot safeguard antibiotic use effectively because people can easily gain access to a wide array of antibiotics including critically important antibiotics.^19^ Thus, antibiotic reclassification was prioritized as the first step to controlling antibiotic distribution. Antibiotic reclassification aims to ensure access to antibiotics when needed, while preventing excessive use of antibiotics by the general public that may accelerate antimicrobial resistance.^20^ Under this principle, several antibiotics should be reclassified as prescription-only drugs, while some would remain as pharmacist-dispensed drugs.

Box 3 **Regulation of antimicrobial drug distribution: Strategy 2 of Thailand’s national strategic plan on antimicrobial resistance 2017–2022**

**Goal**

20% reduction in antimicrobial drug consumption in humans.

**Strategy**

Regulation of antimicrobial drug distribution.

**Achievements**

In the human sector:

- Withdrawal from the market of inappropriate antibiotics (such as oral colistin and antibiotic lozenges for sore throat).
- Implementing the three-phase antimicrobial reclassification plan. Phase 1 was completed in 2019: antituberculosis drugs and all antibiotics for injection are now prescription drugs. Phase 2 is ongoing in 2021. This phase is the most difficult because it covers all oral antibiotics and affects a wide group of stakeholders such as pharmaceutical companies, pharmacies and patients. We developed a reclassification diagram to help the stakeholders to understand the criteria and procedures used in a reclassification process. The tool is based on local evidence and the World Health Organization Access, Watch and Reserve classification of antibiotics.^17^ Phase 3, which covers antibiotics for topical use and external use, will follow in 2023.

In the animal sector:

- Reclassifying penicillins, quinolones, cephalosporins, macrolides, polymyxins and medicated premix to be prescription-only drugs, for prescription by a veterinarian.
- Resolving an ambiguity of medicated feed regulation. Previously, it was unclear whether production of medicated feed should be regulated under the Drug Act or the Animal Food Quality Control Act. Later, a decision was made that Thailand’s Food and Drug Administration regulates medicated premix as a prescription drug under the Drug Act of 1967. The Department of Livestock Development regulates the production of medicated feed from medicated premix under the Animal Food Quality Control Act.
- Prohibiting the use of antibiotics as growth promoters and the registration of antibiotics with an indication for growth promotion.

In the human and animal sectors:

- Developing a surveillance system to monitor national consumption of antimicrobials for humans and animals. Initially a pilot project, the system is now integrated into the routine Food and Drug Administration system.

**Strengths**

- Existence of two foundation laws (the Drug Act and the Animal Food Quality Control Act) that enable ancillary laws to be issued to support Strategy 2 interventions.
- Joint approach of Thailand’s national strategic plan on antimicrobial resistance and the national drug policy.
- Strong collaboration between Thailand’s Food and Drug Administration and the Department of Livestock Development.

**Challenges**

- Resistance from opponents who disagree with antibiotic reclassification.
- Risk of emergence of a black market to supply illegal antibiotics if new regulations to control antibiotic distribution are enforced while the demand for antibiotics is still high.

**Ways forward**

- Monitoring law enforcement and linking with Strategy 5 to improve public understanding about antimicrobial resistance and antibiotic use to ensure an effective implementation of the new regulation.
- Identifying and monitoring the types and amount of human antibiotics that are used in the non-human sector.
- Extending the scope of Thailand’s system for surveillance of antimicrobial consumption to capture antimicrobial use at facility level including hospitals, clinics, pharmacies and farms.

Major challenges to the change were objections from pharmaceutical companies and retailers who feared reduced profits from sales, disagreements among health professionals, and patients’ concerns that they might not be able to access antibiotics or need to pay for doctors’ fees to obtain access to antibiotics.^20^ After examining Thailand’s failed attempt to reclassify antibiotics in 2016 and taking into account the scientific, economic and societal concerns,^13^ we developed an antibiotic reclassification algorithm to provide clear explanations for relevant stakeholders. Reclassification is still ongoing, with some initial successes.

Meanwhile, we developed the system for surveillance of antimicrobial consumption to monitor trends of antimicrobial consumption in humans and animals.^11^ The current system captures data at the national level so it needs to expand its scope to capture antimicrobial use in hospitals, clinics, pharmacies and farms. 

### Resistance control in the human sector

Longstanding policy support by the health ministry and two public organizations responsible for universal health coverage and for hospital accreditation^21^^–^^23^ means that Thailand has been able to reduce the rates of unnecessary use of antibiotics in ambulatory care settings.^23^ An important element of these policies was derived from the Antibiotic Smart Use project – an action research initiative to promote rational use of antibiotics introduced in 2007 by Thailand’s Food and Drug Administration.^24^ However, although antimicrobial resistance in the inpatient setting is more serious and complex, the topic has previously received inadequate policy support. Thus Strategy 3, infection prevention and control and antimicrobial stewardship, was an important component of the national strategic plan (Box 4).

Box 4 **Antimicrobial resistance containment in the human sector: Strategy 3 of Thailand’s national strategic plan on antimicrobial resistance 2017–2022**

**Goals**

50% reduction in antimicrobial resistance morbidity; 20% reduction in antimicrobial drug consumption in humans.

**Strategy**

Infection prevention and control and antimicrobial stewardship in humans.

**Achievements**

In ambulatory care settings:

- Integrating the application of Thailand’s Antibiotics Smart Use project for reducing unnecessary use of antibiotics in upper respiratory tract infection, acute diarrhoea and uncomplicated wounds.^24^ This project was action research initiated by Thailand’s Food and Drug Administration in 2007. It was first adopted by a pay-for-performance policy of the National Health Security Office in 2009 and later integrated into the rational drug use initiative under the national drug policy in 2015. This record demonstrates longstanding policy support for reducing unnecessary use of antibiotics in Thailand.
- Reducing antibiotic prescribing rates from 43.5% to 22.1% (out of 3 087 582 and 5 098 334 outpatient visits, respectively) in upper respiratory infection; from 45.7% to 19.3% in acute diarrhoea (out of 624 452 and 1 251 650 outpatient visits, respectively); and from 68.4% to 45.6% (out of 1 330 707 and 2 506 235  outpatient visits, respectively) in uncomplicated wounds over 2014–2019.^20^

In acute care settings:

- Classifying health-care-associated infections as a communicable disease to be included in surveillance via Thailand’s Communicable Disease Act.
- Endorsing the integrated antimicrobial resistance management initiative^25^ as a framework to address antimicrobial resistance in hospitals.
- Establishing a national coordinating mechanism for the implementation of integrated antimicrobial resistance management in hospitals, led by the Department of Medical Services and the Office of the Permanent Secretary of Public Health.
- Implementing the integrated antimicrobial resistance management initiative in 121 government hospitals. Developing a joint external evaluation tool for integrated antimicrobial resistance management in hospital as a supporting tool for government and nongovernment hospitals to address antimicrobial resistance effectively.
- Updating the national guidelines on antimicrobial resistance surveillance and infection prevention and control. Developing an antimicrobial stewardship guideline and an evaluation tool for the integrated antimicrobial resistance management in hospitals initiative. Conducting training for health professionals such as doctors, infection control nurses, clinical pharmacists and microbiological laboratory staff.
- Conducting a situation analysis on human resources required for addressing antimicrobial resistance in hospitals to explore the status, distribution and challenges of health professionals in hospitals, especially, infectious disease doctors, infection control nurses, clinical pharmacists, medical technicians and microbiological laboratory staff, and to propose policy recommendations to resolve challenges posed to human resources on antimicrobial resistance.

**Strengths**

- Existence of a strong health-care system with universal health coverage.
- Existence of strong networks of health professionals (infectious disease doctors, infection control nurses, pharmacists and microbiology laboratory staff), health professional councils and organizations.

**Challenges**

- Suboptimal leadership at the national level for effective implementation of integrated antimicrobial resistance management in hospital.
- Delayed implementation of integrated antimicrobial resistance management in hospitals due to the COVID-19 pandemic.
- Insufficiency of health professionals in hospitals to address antimicrobial resistance due to inadequate incentives and career pathways.

**Ways forward**

- Specifying which antimicrobial-resistant pathogens need priority action.
- Rethinking and reframing the implementation of integrated antimicrobial resistance management in hospitals under the continuing disruption of the COVID-19 pandemic.

COVID-19: coronavirus disease 2019.

**Fig. 2:**
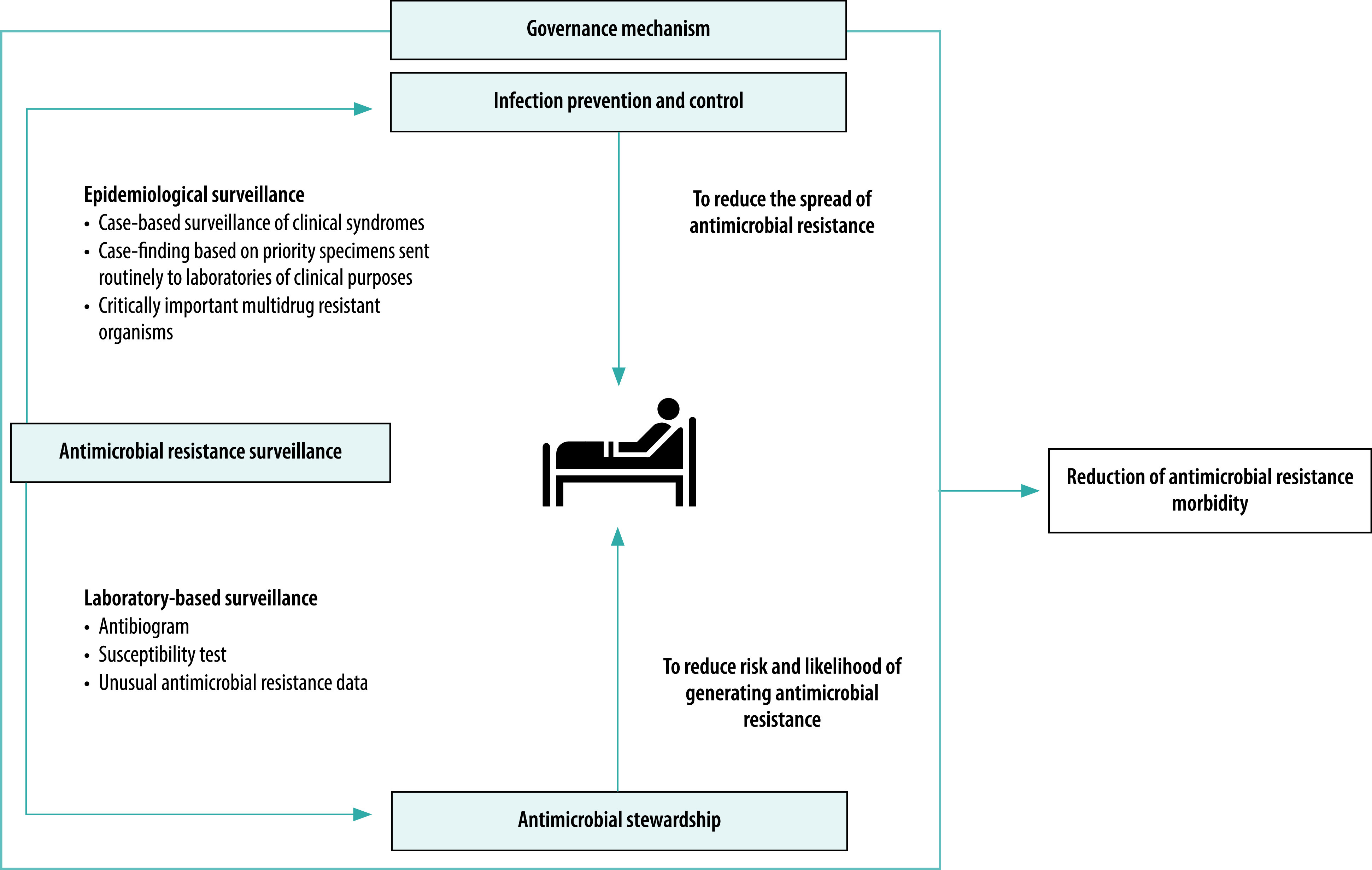
Integrated antimicrobial resistance management in hospital framework in Thailand

Source: Adapted from the subcommittee on reducing antimicrobial resistance impact in hospitals, 2019.^25^

We have created a systems-based, integrated approach to addressing antimicrobial resistance in hospitals (Fig. 2).^25^ The initiative requires organizational leadership with a strong governance mechanism to guide strategic, evidence-based actions to reduce antimicrobial resistance-related morbidity across disciplines, focused on infectious disease doctors, infection control nurses, clinical pharmacists and microbiology laboratory staff. Starting in 2018, the initiative has been launched in 121 government hospitals.^13^ Along with the development of an assessment tool for the systems-based, integrated antimicrobial management in hospitals, the initiative is currently being scaled up nationwide. However, the rate of roll-out of integrated antimicrobial resistance management has not yet fully matched the growing prevalence of antimicrobial resistance in hospitals. The coronavirus disease 2019 (COVID-19) pandemic has also delayed implementation of the initiative in hospitals, as hospital staff were redeployed to respond to the new crisis. Strong leadership at national level is needed to redesign and achieve effective implementation of the initiative within the continuing restrictions of the COVID-19 pandemic. 

### Resistance control in the non-human sector

Strategy 4 of the national strategic plan is antimicrobial resistance prevention and control and antimicrobial stewardship (Box 5), which are relatively new areas of focus for the agriculture and animal sectors in Thailand. We have noted improvements in four main sectors: livestock (especially poultry and swine), aquaculture, companion animals and plants. In the livestock development sector, we focused key interventions on regulatory measures (for example, a requirement for a veterinarian to oversee the medicated feed production with antibiotics) and the initiatives under a public–private partnership (such as strengthening biosecurity and implementing the Raised without Antibiotics initiative^26^^,^^27^ in swine farms).^13^^,^^27^ These interventions are driven by Thailand’s national strategic plan, together with global guidelines and standards.

Box 5 **Antimicrobial resistance prevention and control in agriculture and animals: Strategy 4 of Thailand’s national strategic plan on antimicrobial resistance 2017–2022**

**Goal**

30% reduction in antimicrobial consumption in animals.

**Strategy**

Antimicrobial resistance prevention and control and antimicrobial stewardship in agriculture and animals.

**Achievements**

In the livestock sector:

- Prohibiting the use of antimicrobials as a growth promoter.
- Strengthening regulation on medicated feeds under the Animal Feed Quality Control Act 2015 that requires veterinarians to oversee the medicated feed production.
- Improving the meat-processing systems under the Control of Animal Slaughter and Distribution of Meat Act 2016.
- Partnering with farms and retailers on initiatives such as Raised Without Antibiotics^26^^,^^27^ in 158 swine farms to provide alternative meat products to consumers.^27^

In the aquaculture sector:

- Promoting appropriate use of aquaculture drugs and chemicals, including antibiotics.
- Conducting a series of trainings for aquaculture farmers and regional staff of the Department of Fisheries to strengthen capacity on antimicrobial resistance containment and relevant regulations.
- Investigating the amount of antibiotics used in aquaculture.

In the companion animal sector:

- Developing the guideline for antimicrobial use for companion animals by Thailand’s veterinary dean network, the veterinary council and the university network of One Health approach.
- Investigating the types and amounts of antibiotics used in companion animals.

In crop production:

- Conducting a situation analysis and studies on the effectiveness and impact of antimicrobial use in citrus-greening disease. Investigating antimicrobial residues in citrus fruits.
- Promoting the implementation of the Good Agriculture Practice^28^ in plantations to reduce inappropriate use of antimicrobials in food crop.

**Strengths**

- As a big production base for meat products, especially chicken, food industries in Thailand need to comply with international standards and guidelines such as those from the United Nations Food and Agriculture Organization, the World Organisation for Animal Health and the Codex Alimentarius Commission for food safety standards, as well as the policies of potential partners such as the European Union. This compliance creates an opportunity for improving the quality of meat production for export as well as for domestic consumption.

**Challenges**

- Policy and organizational leadership are required to encourage agencies to expand their roles to address antimicrobial resistance. Antimicrobial resistance is new for the non-human sector, especially for aquaculture, crop production and the environmental sector.
- Despite not being approved by the Department of Agriculture, antibiotics such as ampicillin and amoxicillin are widely used by farmers to treat citrus-greening disease. Antibiotics provide an easier, quicker and cheaper method than the traditional method recommended by the Department of Agriculture (elimination of infected trees, removal of parts of the infected trees and control of vectors for the disease). This issue has not yet been resolved and potential alternatives for antibiotics have yet to be found for greening disease.

**Ways forward**

- Establishing national monitoring systems on antimicrobial consumption in aquatic animals, companion animals and crop production.
- Investing in research to find innovations and alternatives to replace or minimize the use of antibiotics in agriculture.

Fishery biologists, aquatic animal health professionals and veterinarians have jointly developed standards for aquatic animal disease control and prevention, and appropriate use of antimicrobials in aquaculture. The recruitment of veterinarians to the government fisheries department in 2018 was a starting point for Thailand to address antimicrobial resistance and antimicrobial use in the aquaculture sector. Containment of antibiotic use in plants shows slow progress since antibiotics are not legally registered for use in citrus or other plants. Thus, the government agriculture department considers that containment of antibiotic use in plants is beyond its authority. The department does however affirm the utilization of a traditional, non-antibiotic approach to address citrus-greening disease.^13^ In the companion animal sector, an educational approach is a key intervention for promoting appropriate use of antibiotics by veterinarians. 

### Raising public awareness

Strategy 5 was to increase public knowledge on antimicrobial resistance and raise awareness of appropriate use of antimicrobials (Box 6). People often have misconceptions about antibiotics and may be unaware of the problem of antimicrobial resistance.^24^ Past actions to address this issue were run by the food and drug administration and the drug system monitoring and development centre, a civil society organization funded by an autonomous government agency responsible for health promotion. However, addressing public knowledge requires collaboration among a wider array of stakeholders, especially nongovernment sectors such as civil society organizations. We therefore established a joint coordination mechanism among the health ministry and two autonomous government agencies that are responsible for health promotion and participatory public policy, respectively. Implementation is still at an early stage. Specifically, individual agencies have not yet fully integrated their actions or actively engaged other academic^29^^,^^30^ and civil society organization^31^ networks to advance public awareness on antimicrobial resistance. Nevertheless, each agency uses their own networks that are distributed nationwide to disseminate antimicrobial resistance key messages to the public. A strong leadership with abilities to connect ideas to formulate innovative processes for solving complex problems is needed to harmonize agency actions.

Box 6 **Public knowledge on antimicrobial resistance: Strategy 5 of Thailand’s national strategic plan on antimicrobial resistance 2017–2022**

**Goal**

20% increase of public knowledge on antimicrobial resistance and awareness of appropriate use of antimicrobial drugs.

**Strategy**

Public knowledge on antimicrobial resistance and awareness of appropriate use of antimicrobials.

**Achievements**

- Applying a social marketing tactic to encourage the public to avoid taking antibiotics for colds and influenza. The method has been successful in anti-smoking and anti-alcohol campaigns in Thailand. A series of social marketing media developed by the Thai Health Promotion Foundation were broadcast via prime-time television, social media and moving advertisements on transport and reached 9.8 million people within 1.5 months (Social Marketing Department, Thai Health Promotion Foundation, unpublished data, 2020).
- Using the Department of Health service support network of 1 040 000 village health volunteers to spread key messages on antimicrobial resistance to communities.
- Launching Thailand’s first World Antimicrobial Awareness Week, 2020. The campaign has evolved from an Antibiotic Awareness Week run by the Drug System Monitoring and Development Centre since 2013.
- Deploying other methods, including side events at the National Health Assembly, consumer empowerment programmes by the Thai Food and Drug Administration, and using social and unpaid (earned) media to convey shared key messages on antimicrobial resistance.

**Strengths**

- Engagement of the Thai Health Promotion Foundation has elevated antimicrobial resistance communication towards innovative approaches.
- Technical support from Thailand’s National Statistical Office and the International Health Policy Program provides monitoring of public awareness on antimicrobial resistance via the National Health and Welfare Survey, conducted biannually.

**Challenges**

- Despite the integrated operational plan, the interventions of Strategy 5 are mostly activity-based. Strong leadership at the national level is needed to convene multipartner collaborations from other government agencies (such as the education ministry); academia (such as the Mahidol–Oxford Tropical Medicine Research Unit and the Anti-Microbials in Society Hub); and nongovernmental or civil society organizations (such as Greenpeace–Thailand).

**Ways forward**

- Rethinking and reframing implementation of the strategy to engage a wider array of stakeholders and to ensure actions are integrated.

### Governance mechanisms

Strategy 6 involves governance mechanisms to ensure political leadership and concerted efforts are in place to sustain antimicrobial resistance-related actions (Box 7). This strategy serves as a national focal point in several ways. First, fragmented actions across agencies and sectors can be consolidated through the national governance mechanism and multisectoral collaboration (Box 1). Second, the strategy links global and national policy and actions by working closely with the foreign affairs ministry. Third, the strategy allows progress to be monitored and challenges to be identified in the overall implementation of the national strategic plan and provides essential support and joint action to move specific strategies forward. Finally, by working with the WHO country cooperation strategy we can connect the antimicrobial resistance programme with research institutes and networks to provide technical support for implementation of Thailand’s national strategic plan.

Box 7 **Governance mechanisms: Strategy 6 of Thailand’s national strategic plan on antimicrobial resistance 2017–2022**

**Strategy**

Governance mechanisms to ensure political leadership and concerted efforts are in place to sustain antimicrobial resistance-related actions.

**Achievements**

- Leveraging antimicrobial resistance to high-level visibility and promoting policy advocacy. Launching an event on Thailand’s national strategic plan on antimicrobial resistance by the Prime Minister of Thailand.^13^ Co-signing a call-to-action declaration against antimicrobial resistance, led by the deputy prime minister and 23 organizations.
- Establishing and convening the national governance mechanism for strengthening multisectoral collaboration.
- Monitoring progress and challenges in Strategies 1–6 of the national strategic plan on antimicrobial resistance and assisting the implementation of Strategies 2, 3 and 5.
- Convening national forums on antimicrobial resistance in 2018 and 2020 to reaffirm political commitment, update progress and strengthen multisectoral collaboration domestically and internationally.
- Running the antimicrobial resistance programme with the WHO country cooperation strategy for Thailand. Approximately 20 projects have been approved under the programme to support implementation of the national strategic plan.
- Advocating for global action for addressing antimicrobial resistance, in collaboration with the foreign affairs ministry .
- Conducting research mapping and prioritization on antimicrobial resistance to identify research gaps to support implementation of the national strategic plan.
- Conducting the midterm review of implementation of the national strategic plan, and convening a drafting process for Thailand’s national action plan on antimicrobial resistance 2023–2027.

**Strengths**

- Existence of the antimicrobial resistance programme within the WHO country cooperation strategy to support the implementation of Thailand’s national strategic plan on antimicrobial resistance.
- Ability to connect polices on antimicrobial resistance among global, regional and national levels. Ability to convene multipartner, multisectoral collaboration between high-level policy and operational levels.

**Challenges**

- Maintaining an antimicrobial resistance issue at a high-level policy agenda.
- Vulnerability of the national governance mechanism due to a committee-based approach. Vulnerability of funding after the end of the antimicrobial resistance programme within the WHO country cooperation strategy for Thailand in 2021.

**Ways forward**

- Extending the timeframe of Thailand’s national strategic plan on antimicrobial resistance from 2017–2021 to 2022 so that the next national action plan on antimicrobial resistance (2023–2027) can synchronize with the national strategy (2018–2037).
- Accelerating the overall implementation of Thailand’s national strategic plan on antimicrobial resistance to come closer towards the goals.
- Convening the drafting process of the next national action plan on antimicrobial resistance (2023–2027) through a full engagement of multisectoral stakeholders under the One Health approach.

WHO: World Health Organization.

## Interim results

We have identified five interim results corresponding to the five goals of Thailand’s national strategic plan on antimicrobial resistance.

### Resistance prevalence

According to Thailand’s national antimicrobial resistance surveillance centre, during 2017–2020, the trend of carbapenem-resistant *Acinetobacter* spp. was relatively stable, yet high, around 67–70% (from 20 082 out of 29 795 to 14 392 of 20 542 isolates). In contrast, resistance of carbapenem-resistant Enterobacteriaceae, especially meropenem-resistant *Klebsiella pneumoniae*, increased significantly from 8.6% to 11.5% (from 3510/40 814 to 2816/24 081 isolates) over the same time period.^32^ These findings reflect the need to accelerate the effective implementation of Strategy 3 for addressing antimicrobial resistance in hospitals. The trend of penicillin-non-susceptible *Streptococcus pneumoniae* from blood samples decreased from 37.8% to 18.8% (from 822/2174 to 45/240 isolates),^32^ which might be a result of a success of continuing efforts to reduce unnecessary use of antibiotics in upper respiratory tract infection in ambulatory care settings. The prevalence of extended spectrum β-lactamase producing *Escherichia coli* slightly decreased from 50% to 46% (from 27 287/54 558 to 9327/20 285 isolates) in humans.^32^ The prevalence of antimicrobial resistance in the non-human sector is unknown at the country level because data are scattered. This situation highlights the need for robust antimicrobial resistance stewardship, in tandem with integrated surveillance systems under the One Health approach.

### Antimicrobial use in humans

Data from Thailand’s working group on surveillance of antimicrobial consumption^11^ indicates that during 2017–2019, antimicrobial consumption in humans increased by 20.9% (from 68.7 to 83.0 defined daily dose per 1000 inhabitants per day),^33^ compared with the goal of 20% reduction. Potential explanations are twofold. First, there may be increased antimicrobial use in the human sector as indicated by the increasing trends of carbapenem-resistant Enterobacteriaceae. Second, human antibiotics are widely used in agriculture and companion animals (see Strategy 4) and the volume of their use remains unknown. To reduce antimicrobial consumption in humans, we need to accelerate implementation of the integrated antimicrobial resistance management in hospital strategy. To do this we need to strengthen the infection prevention and control and antimicrobial stewardship (Strategy 3); control antibiotic distribution (Strategy 2); and investigate the situation and ensure appropriate use of human antibiotics in the non-human sector (Strategy 4).

### Antimicrobial use in animals

During 2017–2019, antimicrobial consumption in animals decreased by 49% (from 658.73 to 336.25 mg per population correction unit for Thailand),^33^ exceeding the target goal of a 30% reduction. The success can be attributed to several factors. First, Strategy 4 focuses on large agricultural food production and processing industries that account for an estimated 70–80% of the market share in Thailand. Second, a new regulation requires a veterinarian to be present at factories to oversee the production of animal feed medicated with antibiotics before the feed is delivered to farms. This ruling prevents farmers from self-preparing medicated feed. Finally, the strategy generates a niche market for the food industry to provide alternative meat products to consumers, for example within the Raised without Antibiotics initiative.^27^

### Public awareness-raising

Thailand’s national health and welfare survey^12^ revealed a slight improvement in public awareness of antimicrobial resistance and appropriate use of antibiotics in 2017 and 2019 (from 23.7% out of 27 762 respondents^11^ versus 24.3% out of 27 900 respondents, respectively).^33^ This result reflects the need to accelerate an effective implementation of Strategy 5, especially to widen an array of stakeholders and harmonize their actions to avoid duplication of action and to communicate effectively with the public.

### Country capacity

In 2017 we made a baseline assessment on country capacity to address antimicrobial resistance using the joint external evaluation tool for International Health Regulations (2005).^34^ The results indicated that Thailand has a demonstrated capacity in antimicrobial resistance detection with laboratory-based surveillance; a developed capacity in infection prevention and control and surveillance of infections caused by antimicrobial resistance; and a limited capacity in antimicrobial stewardship activities including antimicrobial regulation.^35^ In 2019, the Global Health Security Index ranked Thailand 22nd globally on antimicrobial resistance management.^36^ We hope that the country’s capacity on antimicrobial resistance management will reach the national goal in the 2022 joint external evaluation assessment.^37^

## Lessons learnt

Four key lessons learnt are worth mentioning. First, elevating antimicrobial resistance to high-level visibility and establishing the national governance mechanism should be done at an early stage to galvanize and organize the multisectoral collaborations that lead to effectively consolidate actions under the One Health approach.

Second, the pace of implementation varies across organizations depending on how they prioritize antimicrobial resistance against other issues; how they perceive the relevance and ownership of Thailand’s national strategic plan; and how capable they are of convening multipartner, multisectoral collaborations and embedding the strategic plan’s actions into their workflow. Missing any of these factors impedes implementation.

Third, investment in building the monitoring and evaluation platform should go in parallel with implementation. Strenuous efforts are needed, especially for countries such as Thailand whose databases are fragmented or not in place. Notably, including the antimicrobial resistance programme within the WHO country cooperation strategy for Thailand makes the establishment of a monitoring and evaluation system possible even when the government fiscal budget is inadequate (and usually prioritized for interventions).

Finally, although Thailand’s national goals may be over-ambitious, the goals stimulate actions that go beyond expectations. Indeed, discrepancies between the plan and reality are not unusual as the implementation phase often confronts with dynamic and unpredictable circumstances, not just the goals alone. Thus, we argue that goals that are measurable, despite being highly ambitious, can accelerate action and overcome limitations.

## Challenges

Several major challenges can be noted. First, the current political commitment towards tackling antimicrobial resistance in Thailand seems to be weaker than in the development phase of the national strategic plan. The drift could be attributed to several reasons,^38,39^ including the COVID-19 pandemic. To maintain political commitment, evidence-based policy communication including evidence of success^40^ is essential to keep high-level leaders involved. Additionally, a country’s active engagement with the global policy provides mutually positive momentum for global and national actions in addressing antimicrobial resistance.^9^

Second, the current governance structure of the action plan on antimicrobial resistance is committee-based and therefore relies heavily on the secretary teams and a group of committed persons to drive the national strategic plan forward. Without a redesigned governance structure, the implementation process could be vulnerable to implementation fatigue or loss of skilful staff due to retirement or moving jobs.

Third, funding is available, but fluctuating. Implementation of the national strategic plan is mainly supported via the government fiscal budget and funding from donors. An example of secured funding from donors is the country cooperation strategy for Thailand for which WHO, the health ministry and three public health agencies agreed to contribute a non-earmarked, pooled fund for the antimicrobial resistance programme. In contrast, despite being a regular source, the government fiscal budget fluctuates annually because the budget allocation to projects depends on an agency’s justification in different fiscal years. Thus, securing stable, adequate funds to support long-term efforts to address antimicrobial resistance is important.

Fourth, a problem of data fragmentation makes it difficult to obtain a comprehensive understanding of antimicrobial resistance and antimicrobial use within and across sectors. Progress in synchronizing relevant data has been noted (see Strategies 1 and 2) but long-term investment is still needed to advance and maintain the database alignment.

Fifth, the COVID-19 pandemic has had mixed effects. Large amounts of financial and human capital have been diverted to management of the pandemic, resulting in delayed implementation of Thailand’s national strategic plan on antimicrobial resistance. In addition, in some countries the COVID-19 pandemic has increased the use of antibiotics in hospitals.^41^ On the other hand, the pandemic strengthens infection prevention and control in hospitals and disease prevention in the community through mask wearing and hand cleaning. Fewer cases of respiratory infections in Thailand have been noted in 2020,^42^ possibly leading to lower use of antibiotics.^43^ However, further investigation of these findings is needed.

Finally, as a cross-cutting issue, antimicrobial resistance is related to other policies, such as food safety and national drug policy. Duplication of actions sometimes occurs. Policy coherence should be scrutinized to avoid duplication and to increase the efficiency of processes.^44^ Additionally, global policy coherence is needed to support national actions. For example, our successful engagement with partners in the environmental sector may not be sustained if global policy in addressing antimicrobial resistance in the environment remains conceptual, without concrete actions.

## Ways forward

Thailand’s national strategic plan on antimicrobial resistance successfully consolidates actions under the One Health approach. Significant progress can be seen but many challenges remain. Interim results show success in reduction of antimicrobial consumption in animals. Nevertheless, long-term results need to be monitored. Contributing factors for success include political commitment, multisector collaboration, evidence-based policy guidance and allocated budgets. The impacts of emergent infectious diseases, as highlighted by the COVID-19 pandemic, need to be considered when addressing antimicrobial resistance in the future. In the next stage, we will be discussing the structure of the national governance mechanism, budgeting for the next strategic plan and engaging a wider group of stakeholders to maximize the impact of the plan.
